# Mediating Influences of the Exchange Relationship with a Preceptor on the Relationship between Burnout and Job Retention Intention among New Nurses in Korea

**DOI:** 10.3390/healthcare11182575

**Published:** 2023-09-18

**Authors:** Jihyun Kim, Yaki Yang

**Affiliations:** 1 Department of Nursing, Kunsan College of Nursing, Kunsan-si 54068, Republic of Korea; rnjinny040@kcn.ac.kr; 2 Department of Nursing, Wonkwang University, Iksan-si 54538, Republic of Korea

**Keywords:** nurses, burnout, relationship, intention, preceptorship

## Abstract

The main factor in the turnover of new nurses in Korea is burnout, and a high turnover rate can lead to discontinuation in the nursing profession, due to failure to adapt to the organization. This study aimed to examine the mediating influences of an exchange relationship with the preceptor on the relationship between burnout and job retention intention among new nurses in Korea. Data were collected from 210 new nurses in three general hospitals from 2 May to 30 June 2023. The following statistical analysis were conducted: *t*-test, ANOVA, the Scheffé test, Pearson’s correlation coefficient analysis, and Hayes Process Macro Model 4 (to test the mediating effect). Burnout was negatively associated with job retention intention (r = −0.54, *p* < 0.001) and the exchange relationship with the preceptor (r = −0.29, *p* = 0.001). The exchange relationship with the preceptor was positively associated with job retention intention (r = 0.38, *p* < 0.001). Furthermore, the mediation analysis indicated that the exchange relationship with the preceptor mediated the relationship between burnout and job retention intention. According to the results, the impact of burnout on the job retention intention was mediated by the exchange relationship with the preceptor. Therefore, to increase the job retention intention of new nurses, developing programs to enhance the exchange relationship with the preceptor are recommended.

## 1. Introduction

According to the *2020 Nursing Statistics Yearbook* published by the Korean Nurses Association, the number of nurses working in medical institutions in South Korea is very insufficient, with only 3.8 nurses per 1000 population; this is less than half of the OECD (Organization for Economic Cooperation and Development) average of 8.9 nurses. Nurses, as members of hospital organizations, observe patients’ conditions and provide nursing interventions around the clock. Therefore, the level of nurse availability in acute medical institutions is closely related to reducing patient mortality rates and shortening hospital stays. In 2022, the overall nurse turnover rate in South Korea was reported to be an average of 15.8% [1]. Particularly concerning is the turnover rate of new nurses, which was 52.8%. This means that over half of the newly graduated nurses leave the clinical field shortly after becoming nurses, highlighting the vulnerability of the nursing workforce structure.

The intention to remain in the current nursing position or to discontinue the search for alternative professions can be defined as the job retention intention [2]. Given the high turnover and attrition rates among nurses, there is a pressing need to increase job retention intention in order to address the nursing workforce problem [3]. The short tenure and high turnover rates of nurses can result in the loss of specialized workforce both at the institutional and national levels. In particular, the high turnover rate among new nurses with less than one year of experience not only leads to the discontinuation of nursing roles due to failed organizational adaptation but also necessitates immediate practical solutions for improvement. Understanding the job retention intention can have a more positive impact on retaining nursing personnel within an organization compared to assessing the intention to leave, potentially reducing the costs associated with employee turnover and enhancing the efficiency of workforce management [4].

New nurses embark on their social journey with a mix of excitement, anticipation, and exhilaration for their new career. However, they often experience negative perceptions of their own incompetence due to factors such as lack of knowledge about nursing tasks, insufficient practical skills, and disparities between what they learned in their studies and the realities of their work [5]. To address these challenges, healthcare institutions select and train preceptors based on their clinical experience and practical capabilities, according to their own criteria. Preceptors serve as role models within nursing units, guiding newly appointed nurses to adapt to their new environment, facilitate socialization, provide education, assess outcomes, and offer feedback [6]. The utilization of preceptors in education is regarded as a desirable approach to bridge the gap between theory and practice, enhancing the clinical skills of new nurses [7].

The concept of an exchange relationship is proposed to evaluate the overall interactions between members and their peer groups. It measures how easily and quickly members exchange information and seek assistance, as well as their ability to share ideas and feedback [8]. In the process of exchange relationships, when leaders provide opportunities for task execution to members and they positively accept these opportunities, trust is built between the leader and the member. As a result, the member’s emotional connection, characterized by affect, loyalty, contribution, and professional respect increases, ultimately enhancing the quality of the exchange relationship [9].

Now, in most healthcare institutions, new nurses adapt to the hospital environment and perform nursing tasks through interactions with their preceptors after joining the organization [10]. Previous studies analyzing the interactions between new nurses and preceptor found that during the preceptor education period, 66.0% of preceptors and 44.7% of new nurses reported experiencing conflicts [11]. Furthermore, a higher level of trust with immediate supervisors was positively associated with higher job retention intention [12]. The exchange relationship between leaders and members has been statistically correlated with nurses’ job retention intention [13]. Therefore, the quality of the exchange relationship between preceptors and new nurses can be considered a significant factor influencing the intention to stay among new nurses.

Burnout refers to the emotional exhaustion, dehumanization, and reduced personal accomplishment experienced by individuals primarily engaged in interpersonal relationships within an organization [14]. New nurses often experience a sense of shock due to the discrepancy between their education at university and the clinical setting, leading to confusion of values, lack of professional knowledge and skills required in the practical field, and inadequate decision-making abilities [15]. This shock is accompanied by excessive workload, complex and challenging interpersonal relationships, role conflicts, and shift work, all contributing to increased stress and burnout among new nurses [16]. The burnout experienced by nurses has been reported as a significant factor negatively influencing their job retention intention [17].

Studies investigating the factors influencing the job retention intention among new nurses have identified various factors related to demographic, personal, and environmental characteristics, as well as job-related aspects. Some of these factors include: realistic shock and coping resilience [18], transition shock, work environment, and self-efficacy [19], nursing practice readiness and organizational socialization [20], and the meaning of work, organizational commitment, and professional self-image [21]. Furthermore, a correlation has been established between burnout and intention to leave among new nurses [22]. Research has also reported that emotional burnout mediates the relationship between burnout and intention to leave among clinical nurses [23]. However, previous studies have conducted separate analyses of the relationships between burnout, the exchange relationship with preceptors, and intention to stay among new nurses. There are limitations in comprehensively understanding the specific mechanism through which the exchange relationship with preceptors mediates the relationship between burnout and intention to stay in the context of burnout and job retention intention.

This study aimed to examine the relationship between burnout, the exchange relationship with the preceptor, and job retention intention among new nurses in Korea, and to identify the mediating influences of the exchange relationship with the preceptor on the relationship between burnout and job retention intention. Through this research, we seek to provide foundational data that can enhance the understanding of factors influencing new nurses’ job retention intention and highlight the significance of the exchange relationship with the preceptor. The four hypotheses of this study were formulated.

**H1.**  *Burnout affects job retention intention among new nurses.*

**H2.**  *Burnout affects the exchange relationship with the preceptor among new nurses.*

**H3.**  *The exchange relationship with the preceptor affects job retention intention among new nurses.*

**H4.**  *The exchange relationship with the preceptor is a mediating influence between burnout and job retention intention among new nurses.*

## 2. Materials and Methods

### 2.1. Study Design and Sample

This study is a descriptive survey study conducted to examine the mediating influences of the exchange relationship with the preceptor in the relationship between burnout and job retention intention among new nurses in Korea.

The participants of this study were selected through convenience sampling from nurses employed in three general hospitals located in J province in Korea who had been working at the hospital for less than 12 months. The required sample size for this study was calculated using G*Power 3.1.9.7 software, with a significance level (α) of 0.05, power of 0.95, effect size of 0.15, and 14 predictor variables. The minimum sample size was determined to be 194 participants. Considering a dropout rate of 10%, a total of 220 questionnaires were distributed. Of these, 215 were collected, and after excluding incomplete and unreliable responses, a final analysis was conducted with 210 completed questionnaires.

### 2.2. Instrument

A structured self-report questionnaire was utilized for data collection, consisting of a total of 49 items: 12 items for general characteristics, 20 items for burnout, 11 items for the exchange relationship with the preceptor, and 6 items for job retention intention. Prior to using the research instruments, all measurements were employed only after obtaining approval for their usage.

#### 2.2.1. Burnout

Burnout was measured using a modified and refined version of the tool developed by Pines, Aronson, and Kafry (1981) [24], which was originally designed for general workers in the service industry, adapted for use with nurses [25]. This measurement consists of a total of 20 items across three subscales: physical burnout, emotional burnout, and mental burnout. Responses were collected using a 5-point Likert scale with ranges from 1 to 5: the higher the score, the higher the burnout. It consists of items such as “I am tired” and “I am depressed”, and the Cronbach’s at the time of development was 0.85. The reliability of each subdomain was 0.78 for physical burnout, 0.72 for emotional burnout, and 0.66 for mental burnout. Cronbach’s was 0.89 in this study, and the reliability of each subdomain was 0.88 for physical burnout, 0.84 for emotional burnout, and 0.71 for mental burnout.

#### 2.2.2. Exchange Relationship between New Nurses and Preceptor

To measure the exchange relationship with the preceptor, a tool adapted [26] from the leader–member exchange (LMX) scale developed by Liden and Maslyn (1998) [8] was used. The original LMX scale was designed for service and industrial members, and its validity was confirmed through adaptation. This measurement consists of a total of 11 items across four subscales: affect, loyalty, contribution, and professional respect. Responses were collected using a 4-point Likert scale with ranges from 1 to 4: the higher the score, the higher the exchange relationship with the preceptor. It consists of items such as “I like the preceptor as a person” and “I am impressed by the preceptor’s job knowledge”, and the Cronbach’s at the time of development was 0.89. Cronbach’s was 0.92 in this study, and the reliability of each subdomain was 0.91 for affect, 0.90 for loyalty, 0.68 for contribution and 0.92 for professional respect.

#### 2.2.3. Job Retention Intention

Job retention intention was assessed using a tool developed by Cowin (2002) [2] and adapted into Korean for use with nurses [27]. This measurement consists of a total of 6 items and the 8-point Likert scale ranges from 1 to 8: the higher the score, the higher the job retention intention. It consists of items such as “I intend to continue working as a nurse” and “I will work as a nurse as long as possible”, and Cronbach’s was 0.97 at the time of development and 0.85 in this study.

### 2.3. Data Collection

This took place at three general hospitals in J province in Korea from 2 May to 30 June 2023. We explained the research objectives and survey procedures to the participants. Those who voluntarily agreed to participate signed and responded to the survey. Response data were collected in opaque envelopes to ensure the confidentiality of personal information. Participants were informed that the response data would be stored for 3 years and then disposed of properly. After completing the survey, coffee coupons were given to the participants in return; the estimated time required for survey completion was around 10–15 min.

### 2.4. Ethical Consideration

This study protocol was approved by the Institutional Research Board of Wonkwang University Hospital, Jeonbuk, South Korea. Before filling out the questionnaire, the researcher explained the purpose of the study to all participants and considered the ethical rights of the participants. The researcher ensured the autonomy, confidentiality, and freedom to withdraw to all participants who voluntarily participated in the study.

### 2.5. Statistical Analysis

Data analysis was conducted using the statistical software SPSS/WIN 28.0 (IBM, Armonk, NY, USA). Descriptive statistics were conducted to evaluate frequencies and percentages for the categorical variables, and central tendency for the continuous variables. Pearson’s r correlations were performed to examine the relationships between continuous variables. To assess any significant differences in the average scores of the job retention intention, *t*-test, and one-way analysis of variance (ANOVA) were performed for each of the categorical independent variables. The significance of the mediating influences was analyzed using Hayes’ SPSS PROCESS macro model no. 4 [28]. The significance test of the indirect effect was performed using bootstrapping of the PROCESS macro with 10,000 samples and a confidence interval of 95.0%. Hayes’s process macro uses bootstrapping, which does not assume normality of the distribution of indirect effects, so it is a method of overcoming the limitation of not reflecting the actual sampling distribution according to the assumption of normality of the Sobel test [28].

## 3. Results

### 3.1. General Characteristics of Participants and Differences in Job Retention Intention According to the General Characteristics

Table 1 displays the general characteristics of participants and the differences in job retention intention according to the general characteristics. Job retention intention differed significantly according to education level (t = −2.28, *p* = 0.026), work unit (F = 4.34, *p* = 0.002), clinical experience (F = 3.91, *p* = 0.021) and the last month’s salary (F = 15.34, *p* < 0.001). Post hoc analysis using the Scheffé test revealed that nurses working in the intensive care unit and emergency room had higher intention to stay compared to those in the medical ward. Additionally, participants with work experience of 5 to 9 months had higher intention to stay than those with 10 to 11 months of work experience.

### 3.2. Degrees of Burnout, Exchange Relationship with Preceptor and Job Retention Intention

Table 2 shows the degrees of the main variables of the study. The participants’ mean scores on burnout, exchange relationship with preceptor and job retention intention were 2.65 (SD = 0.60), 3.51 (SD = 0.45), and 5.70 (SD = 1.07), respectively.

### 3.3. Correlation between Burnout, Exchange Relationship with Preceptor and Job Retention Intention

Table 3 shows the correlations of the main variables of the study. Burnout was negatively associated with job retention intention (r = −0.54, *p* < 0.001) and exchange relationship with preceptor (r = −0.29, *p* = 0.001). The exchange relationship with the preceptor was positively associated with job retention intention (r = 0.38, *p* < 0.001).

### 3.4. Mediating Influences of Exchange Relationship with Preceptor between Burnout and Job Retention Intention

**Figure 1:**
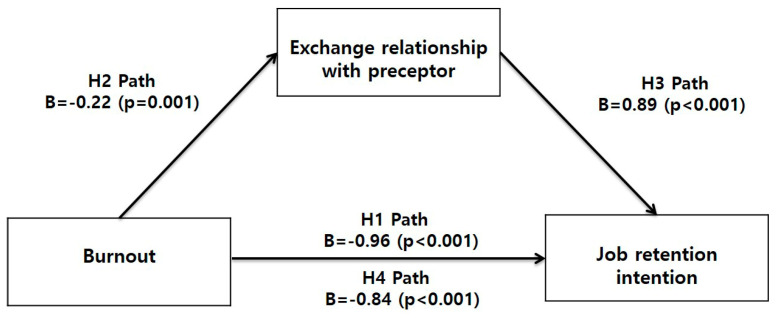
Mediating influences of exchange relationship with preceptor on relationship between burnout and job retention intention.

The results of the mediation analysis are presented in Table 4 and Figure 1. The total effect of burnout on job retention intention was significant (c path: B = −0.96, *p* < 0.001, 95% CI = −1.17, −0.76), after adjusting for education level (1 = college), work unit (1 = medical ward), clinical experience (1 = ≤4 months), and last month’s salary (1 = ≤2.5 million won). Also, the direct effects of burnout on exchange relationship with preceptor (H2 path: B = −0.22, *p* = 0.001, 95% CI = −0.31, −0.19) and exchange relationship with preceptor on job retention intention (H3 path: B = 0.89, *p* < 0.001, 95% CI = 0.29, 0.84) were significant after adjusting for covariates. Moreover, the direct effect of burnout on job retention intention was significant after adjusting for exchange relationship with preceptor and other covariates, (H4 path: B = −0.84, *p* < 0.001, 95% CI = −1.04, −0.64), indicating that exchange relationship with preceptor mediated the association between burnout and job retention intention. Furthermore, the indirect effect of exchange relationship with preceptor was statistically significant (H2 × H3: B = −0.12, 95% bias-corrected bootstrap CI = −0.21, −0.05), suggesting that burnout has an indirect negative effect on job retention intention via exchange relationship with preceptor.

## 4. Discussion

Based on the analysis of the impact of the exchange relationship with the preceptor and burnout on job retention intention among new nurses, this study aimed to examine the mediating influences of the exchange relationship with the preceptor on the relationship between burnout and job retention intention and to provide foundational data for an intervention program to enhance job retention intention among new nurses.

The participants’ exchange relationship with the preceptor had an average score of 3.56 out of 4. This was similar to the findings of a study that assessed the exchange relationship with preceptors among new nurses using the same tool [29]. In this study, the nurses and the preceptors had at least moderate levels of exchange relationships. This circumstance arguably arises from feelings of trust and interactions between nurses becoming acclimated to a new environment and the preceptors. When examined by subdomain, professional respect was the highest, followed by affect, loyalty, and contribution. These results are consistent with a study conducted with new nurses as participants [29]. The reason for the high level of professional respect is that new nurses recognize the expertise of preceptors as they encounter their extensive nursing knowledge and clinical skills in the clinical setting, leading to mutual understanding and strong communication.

The participants experienced burnout with an average score of 2.65 out of 5, indicating at least a moderate level of burnout. This score was lower than the findings of a study [30] conducted with nurses who had started independent work within a year of graduation, and higher than the results of a study that measured burnout among ICU nurses using the same tool [31]. The degree of burnout among new nurses can vary depending on the hospital’s type, size, nursing workforce, and geographical location, indicating the need for repeated studies that take into account hospital variations in size and location. However, the prevalent findings of at least moderate burnout in most studies can be attributed to the inherent nature of nursing work, which is closely tied to patients’ lives and involves considerable stress.

Particularly noteworthy is the finding that the subdomain of physical burnout exhibited the highest level of burnout, which is consistent with research conducted on nurses in general hospitals [32]. The heightened physical burnout can be attributed to the nature of nursing tasks, involving interactions with patients and various departments, diverse and unilateral patient demands, increased workload due to a shortage of nursing staff, and administrative and educational responsibilities, all contributing to physical burnout [33]. Burnout leads to decreased job satisfaction and negatively affects both patients and hospital organizations. Thus, efforts to prevent or reduce burnout are crucial for the professional growth of nurses and the advancement of the nursing profession. Rather than addressing burnout as a personal issue for nurses to solve themselves, administrators need to improve the organizational environment. Diagnosing the organizational work environment accurately and proactively intervening to provide a reasonable organizational structure and appropriate workload should be prioritized. A qualitative study could be proposed to collect specific data to identify factors influencing burnout and prepare alternatives for new nurses.

The participants’ level of job retention intention was 5.70 out of 8 points. This score was higher than the findings of studies that measured the job retention intention among new nurses using the same tool [34,35], and lower than the job retention intention among new nurses with less than 6 months of clinical experience in a study conducted on university hospital nurses [36]. The variations in results across different studies suggest that the level of job retention intention can differ based on various factors such as hospital size and geographical location, highlighting the need for repeated studies.

The job retention intention among the participants varied, based on their general characteristics, particularly in terms of their education level, work unit, clinical experience and the last month’s salary. Post hoc analysis revealed that nurses working in the intensive care unit (ICU) had a higher job retention intention compared to those in the internal medicine ward. In the hospital where the participants belong, due to COVID-19 in recent years, patients from various departments are hospitalized in the internal medicine ward, and in the intensive care unit there are only patients from the relevant. It is believed that new nurses assigned to the internal medicine ward would have had more difficulty adapting to work. Additionally, nurses with 5–9 months of work experience expressed a higher intent to continue working than those with 10–11 months of experience. These findings partially align with the results of a study [35] which showed significant differences in job retention intention based on factors such as age, marital status, educational background, clinical experience, current department experience, current department assignment, work schedule, and desired department placement. However, the results diverged from a study [34] which indicated statistically significant gender-based differences.

Factors influencing nurses’ job retention intention include various individual characteristics and work-environment-related aspects, as demonstrated by prior research on factors like motivations for choosing a nursing career and salary levels [37]. The variability in job retention intention can be attributed to factors such as individual characteristics and work environment. It is worth noting that previous studies have consistently shown that new nurses tend to exhibit lower intent to continue working compared to their more experienced counterparts [20,35]. This suggests that new nurses may not be committed to a long-term nursing career and might be exploring other job opportunities or career paths. Therefore, efforts to enhance nurses’ job retention intention should focus on actively incorporating the perspectives of new nurses, improving the work environment, and elevating overall job satisfaction within the nursing profession.

The perceived exchange relationship with the preceptor, burnout, and job retention intention among new nurses were found to be correlated. Specifically, the relationship with the preceptor was negatively correlated with burnout and positively correlated with job retention intention. In other words, a higher level of exchange relationship with the preceptor was associated with lower burnout and higher job retention intention among new nurses. Conversely, burnout showed a negative correlation with job retention intention, indicating that lower burnout was associated with higher job retention intention. These findings are consistent with a study conducted on university hospital nurses, which also reported a negative correlation between burnout and job retention intention [17].

Similarly, a study investigating the leader–member exchange relationship and turnover intention among nurses in general hospitals found a negative correlation between the exchange relationship and turnover intention [9]. This suggests that as the exchange relationship increases, turnover intention decreases. Therefore, promoting mentoring activities between experienced nurses and new nurses and implementing preceptorship programs based on understanding the preceptor’s mentoring style and the characteristics of new nurses could help facilitate positive interpersonal relationships and consequently enhance new nurses’ job retention intention.

Lastly, this study confirmed the mediating influences of the exchange relationship with preceptors on the relationship between burnout and job retention intention. While it is challenging to find direct comparative studies that investigate the mediating influences of the exchange relationship with the preceptor on the relationship between burnout and job retention intention among new nurses, this is similar to the results showing that coaching, a sub-area of the nursing unit manager’s empowering leadership, had a significant effect on new nurses’ job retention intention [20]. Coaching is when a leader helps members handle their duties smoothly [38]. When a new nurse asks for help in a difficult situation, the nursing unit manager provides encouragement, listening, and participation in decision-making. Providing appropriate help, such as opportunities for the reflection of opinions, would have helped them adapt to the organization and increased their job retention intention. As a way to improve the quality of the exchange relationship between new nurses and the preceptor, we suggest including coaching and communication skills when organizing a preceptor education program.

In this study, we have gone beyond the previous findings [23,39,40] that indicated that a positive exchange relationship with the preceptor can prevent burnout among new nurses. We have demonstrated that the exchange relationship with the preceptor plays a mediating role in the relationship between burnout and job retention intention among new nurses. By maintaining a positive exchange relationship with preceptors, new nurses can adapt to the hospital’s regulations and culture and achieve clear role acquisition, job mastery and job satisfaction, thereby increasing their intent to continue working. Consequently, it is essential to establish various programs to enhance the quality of the relationship between preceptors and new nurses. Furthermore, broader follow-up studies incorporating diverse variables are needed to fully comprehend the concept of the exchange relationship between new nurses and preceptors.

This study is significant in examining the relationships among burnout, the exchange relationship with the preceptor, and job retention intention among new nurses. Moreover, it emphasizes the importance of the exchange relationship with the preceptor in the relationship between burnout and job retention intention among new nurses. There is a workplace bullying (Taeum) culture in Korean nursing organizations. This is a slang term derived from the phrase “burn until it turns to ashes”. It usually means that after a new nurse joins a hospital, a senior nurse commits violence against an inexperienced new nurse by using inappropriate teachings. This “Taeum” in clinical settings is a social problem that can lead to life-threatening behavior and also affects maladaptation at work and the turnover of nurses [41]. Based on the results of this study, within the process of operating a preceptorship to help new nurses’ clinical adaptation, there is a need to develop programs that enhance the quality of the exchange relationship between the preceptor and new nurses to increase job retention intention and explore specific strategies to reduce burnout.

However, it is important to acknowledge the limitations of this study. The research was conducted with a convenience sample of new nurses from a single province, which may restrict the generalizability of the results. It is necessary to expand the study’s scope to include a wider range of participants and settings. Additionally, given that the working unit emerged as a key factor influencing burnout and job retention intention in this study, future research could use it as an independent variable to further investigate its impact on burnout and job retention intention among new nurses, through repeated studies. In addition, this study has the limitation that it only dealt with the exchange relationship with the preceptor as perceived by new nurses. There is a need to conduct research that includes the exchange relationships with new nurses as perceived by preceptors.

## 5. Conclusions

The results of this study reveal statistically significant correlations among burnout, the exchange relationship with the preceptor, and job retention intention among new nurses. Moreover, the study indicates that the exchange relationship with the preceptor acts as a mediator in the relationship between burnout and job retention intention. Based on these results, in order to increase the job retention intention of new nurses, there is a need to identify the causes of burnout and provide interventions to reduce it. It is also suggested that further research should be conducted through repeated studies, focusing on different work departments.

To improve the job retention intention among new nurses, strategies to enhance the quality of the exchange relationship with preceptors should be explored. Given the limited existing research on the exchange relationship with the preceptor, there is a need for subsequent studies to delve deeper into this aspect.

## Figures and Tables

**Table 1:** General characteristics of participants and differences in job retention intention according to the general characteristics (*n* = 210).

Characteristics	Categories	*n* (%)	Mean ± SD	T or F	*p* Scheffé
Age (years)	≤23	98 (46.7)	5.59 ± 0.97	0.96	0.384
	24–25	55 (26.2)	5.80 ± 1.17		
	≥26	55 (27.1)	5.79 ± 1.14		
Gender	Men	35 (16.7)	5.80 ± 1.05	0.62	0.535
	Women	175 (83.3)	5.68 ± 1.08		
Marital state	Unmarried	205 (97.6)	5.68 ± 1.08	−1.69	0.093
	Married	5 (2.4)	6.50 ± 1.12		
Education level	College	33 (15.7)	5.42 ± 0.67	−2.28	0.026
	University	177 (84.3)	5.75 ± 1.13		
Religion	Yes	80 (38.1)	5.77 ± 1.10	0.75	0.452
	No	130 (61.9)	5.65 ± 1.06		
Working unit	Medical ward	49 (23.3)	5.25 ± 0.83	4.34	0.002
	Surgical ward	54 (25.7)	5.60 ± 0.99		
	Intensive care unit	55 (26.2)	5.90 ± 1.07		
	Emergency room	35 (16.7)	6.11 ± 1.27		
	Outpatient services	17 (8.1)	5.85 ± 1.13		
Clinical experience (months)	≤4	23 (11.0)	5.59 ± 1.28	3.91	0.021
	5~9	53 (25.2)	6.05 ± 1.30		
	10~11	134 (63.8)	5.58 ± 0.90		
Duration of preceptorship (weeks)	≤4	5 (2.4)	5.63 ± 0.63	0.07	0.978
	5~7	98 (46.7)	5.68 ± 1.01		
	8	45 (21.4)	5.76 ± 1.31		
	9~12	52 (29.5)	5.70 ± 1.07		
Type of work	Shift work	189 (90.0)	5.68 ± 1.06	−0.95	0.344
	Fixed work	21 (10.0)	5.91 ± 1.17		
Desire for working unit	Yes	119 (56.7)	5.71 ± 0.95	0.19	0.850
	No	91 (43.3)	5.69 ± 1.22		
Past practice experiences in working unit	Yes	93 (44.3)	5.71 ± 1.08	0.08	0.940
	No	117 (55.7)	5.70 ± 1.07		
Last month’s salary (million earned)	≤2.5	17 (8.1)	6.99 ± 1.26	15.34	<0.001
	2.6~2.9	35 (16.7)	5.49 ± 0.92		
	≥3	158 (75.2)	5.61 ± 1.00		

**Table 2:** Degrees of burnout, exchange relationship with preceptor and job retention intention (*n* = 210).

Variables	Subscale	Mean ± SD	Min~Max	Range
Burnout	Total	2.65 ± 0.60	1.40~4.25	1~5
	Physical exhaustion	3.48 ± 0.83	1.33~5.00	1~5
	Emotional exhaustion	2.20 ± 0.70	1.00~4.14	1~5
	Mental exhaustion	2.39 ± 0.65	1.00~4.14	1~5
Exchange relationship with preceptor	Total	3.51 ± 0.45	1.64~4.00	1~4
	Affect	3.56 ± 0.55	1.00~4.00	1~4
	Loyalty	3.51 ± 0.60	1.00~4.00	1~4
	Contribution	3.18 ± 0.62	1.50~4.00	1~4
	Professional respect	3.67 ± 0.46	2.00~4.00	1~5
Job retention intention		5.70 ± 1.07	3.33~8.00	1~8

**Table 3:** Correlation between burnout, exchange relationship with preceptor and job retention intention (*n* = 210).

	Burnout	Exchange Relationship with Preceptor	Job Retention Intention
	r (*p*)		
Burnout	1		
Exchange relationship with preceptor	−0.29 (<0.001)	1	
Job retention intention	−0.54 (<0.001)	0.038 (<0.001)	1

**Table 4:** Mediating influences of exchange relationship with preceptor between burnout and job retention intention (*n* = 210).

Path	B	SE	t	*p*	95% CI		R^2^	F(*p*)
					LLCI	ULCI		
Burnout → Job retention intention (H1 path)	−0.96	0.10	−9.30	<0.001	−1.17	−0.76	0.29	86.53 (<0.001)
Burnout → Exchange relationship with preceptor (H2 path)	−0.22	0.05	−4.36	0.001	−0.31	−0.19	0.08	19.03 (<0.001)
Exchange relationship with preceptor → Job retention intention (H3 path)	0.89	0.15	5.83	<0.001	0.29	0.84		
Burnout → Job retention intention (H4 path)	−0.84	0.10	−7.33	<0.001	−1.04	−0.64	0.35	54.65 (<0.001)
Indirect effect	coefficient = −0.12, bias-corrected bootstrap SE = 0.04, 95% bias-corrected bootstrap CI (−0.21, −0.05)							

B: unstandardized coefficients; SE: standard error; LLCI: lower level of confidence interval; ULCI: upper level of confidence interval; adjusted for education level (1 = college), work unit (1 = medical ward), clinical experience (1 = ≤4 months), and last month’s salary (1 = ≤2.5 million won).

## Data Availability

Not applicable.
